# Impact of financial toxicity on adults with cancer during the COVID-19 pandemic: an integrative review

**DOI:** 10.1590/0034-7167-2024-0078

**Published:** 2024-08-30

**Authors:** Luciana de Alcantara Nogueira, Cristiano de Oliveira Ribeiro, Leonel dos Santos Silva, Yasmin Hiorrana dos Santos, Luciana Puchalski Kalinke

**Affiliations:** I Universidade Federal do Paraná. Curitiba, Paraná, Brazil

**Keywords:** Financial Stress, Coronavirus Infections, COVID-19, Neoplasms, Review, Estrés Financiero, Infecciones por Coronavirus, COVID-19, Neoplasias, Revisión

## Abstract

**Objectives:**

to identify the repercussions of financial toxicity on the lives of adult cancer patients during the COVID-19 pandemic.

**Methods:**

an integrative review was conducted using the PubMed, Web of Science, Scopus, and Embase databases, as well as the Virtual Health Library portal, in March 2023.

**Results:**

out of 62 studies found, 13 were included for analysis. The primary repercussions of financial toxicity included difficulties in covering basic expenses such as food, housing, medication, transportation, and internet access; increased anxiety and concerns related to health and financial situations; reduction or absence of income; challenges in obtaining treatment or accessing healthcare services; rising expenses; and telemedicine as a less burdensome alternative.

**Conclusions:**

the pandemic has exacerbated financial toxicity; therefore, healthcare teams must recognize it as an adverse event of oncological treatment and understand its potential to affect various aspects of patients’ lives.

## RESUMEN

**Objetivos:**

identificar las repercusiones de la toxicidad financiera en la vida de pacientes adultos con cáncer durante la pandemia de COVID-19.

**Métodos:**

revisión integrativa realizada en las plataformas de datos PubMed, *Web of Science,* Scopus, Embase y en el portal de la Biblioteca Virtual en Salud en marzo de 2023.

**Resultados:**

de 62 estudios encontrados, 13 fueron incluídos para análisis. Las principales repercusiones de la toxicidad financiera fueron: dificultades para cubrir gastos básicos como alimentación, vivienda, medicamentos, transporte e internet; aumento de la ansiedad y preocupaciones relacionadas con la salud y la situación financiera; reducción o ausencia de ingresos; dificultad para obtener tratamiento o acceso a los servicios de salud; aumento de los gastos y telemedicina como alternativa menos costosa.

**Conclusiones:**

la pandemia ha intensificado la toxicidad financiera; por lo tanto, los equipos de salud necesitan reconocerla como un evento adverso del tratamiento oncológico y comprender que puede afectar diferentes ámbitos de la vida de los pacientes.

## INTRODUCTION

Financial toxicity is an adverse event associated with costly diseases like cancer. It arises from the financial burdens that patients face due to the costs of diagnosis, including tests, medications, transportation, and other related expenses. This situation exposes patients to psychological stress, incorporating financial worries and concerns about health insurance coverage when available. Such challenges can lead to neglect, delays, or reductions in necessary care^(1-5)^.

Among chronic non-communicable diseases, cancer bears the highest treatment costs. This fact is underscored by a study conducted in the United States (US)^(6)^, a country where most research on the costs of cancer drugs is performed. The study revealed that the average monthly treatment with cetuximab®, used for lung cancer, costs $80,000, which translates to $960,000 per year to extend a patient’s life, potentially causing or exacerbating financial toxicity.

Another contributing factor to increased financial toxicity was the Coronavirus Disease 2019 (COVID-19) pandemic, which precipitated a global economic crisis, reduced incomes, and necessitated the adoption of different behavioral rules affecting the entire population. In this context, patients weakened by diagnosis and treatment may experience heightened effects of financial toxicity. They now live with the fear of virus infection, compounded by a lack of resources due to increased costs for food, medication, and the necessities of social isolation, among other issues, which can exacerbate conditions like depression and anxiety.

Research on financial toxicity at the national level is still in its early stages, particularly during the COVID-19 pandemic period. However, studies^(7-8)^ published in 2020 highlight the impacts of the economic crisis associated with the pandemic, emphasizing unemployment, bankruptcy, and other factors that may affect the Gross Domestic Product (GDP) in the medium to long term and exacerbate difficulties in funding cancer treatment. A recent Brazilian editorial^(9)^ highlighted the observation that the pandemic created an unprecedented crisis, where the population found itself in widespread social and economic vulnerability, compounded by the collapse of the Unified Health System (SUS), resulting in financial strain on patients to cover treatment expenses.

In the US, a leader in discussions on financial toxicity, the financial burdens during the pandemic have impacted health service utilization and restricted access to healthcare, with the most severe effects observed among women^(10)^. Given the contemporaneity of the COVID-19 pandemic, there has been limited discussion in Brazil and other countries about the repercussions of financial toxicity on cancer patients, making this topic innovative and significant to the academic community.

## OBJECTIVES

To identify the repercussions of financial toxicity on the lives of adult cancer patients during the COVID-19 pandemic.

## METHODS

This integrative review was structured in six steps: 1) identification of the topic and formulation of the research question; 2) determination of the sample and inclusion criteria; 3) specification of the information to be extracted from the studies; 4) analysis of the results; 5) interpretation of the results; 6) presentation of the findings^(11)^.

The mnemonic PCC was utilized, comprising P (Population) – adult cancer patients; C (Concept) – financial toxicity; C (Context) – the COVID-19 pandemic. Based on these definitions, the research was guided by the question: What are the repercussions of financial toxicity on adult cancer patients during the COVID-19 pandemic as reported in the literature?

The search for studies was conducted in March 2023 across several databases: the National Library of Medicine (PubMed), Web of Science, Scopus, Embase, and the Virtual Health Library (BVS). Descriptors from the Health Sciences Descriptors (DeCS) and Medical Subject Headings (MeSH) platforms included: “financial toxicity,” “financial stress,” “neoplasms,” and “COVID-19,” along with their alternative terms “cancer” and “financial toxicity.” These terms were combined using the Boolean operators AND and OR (Chart 1).

**Chart 1:** Search Strategy Used, Curitiba, Paraná, Brazil, 2023

Consulted Databases and Search Strategy Employed	
Pubmed	“financial toxicity” OR “financial stress” AND “neoplasms” AND “covid-19”
Web of Science	“financial toxicity” OR “financial stress” AND “cancer” AND “covid-19”
Scopus	“financial toxicity” OR “financial stress” AND “neoplasms” AND “covid-19”
Embase	“financial toxicity” OR “financial stress” AND “neoplasms” AND “covid-19”
*BVS*	*“toxicidade financeira” AND “câncer” AND “covid-19”*

Included were open access studies that specifically addressed the repercussions of financial toxicity, published from the onset of the pandemic (2020) through March 2023, and were either quantitative or qualitative, in Portuguese, English, or Spanish. Excluded were texts categorized as comments, editorials, letters to the editor, and pre-prints.

The search strategy and the descriptors used are detailed in Chart 1.

The selection of articles was carried out by two independent researchers to mitigate selection bias. Any disagreements were discussed and assessed collaboratively, and with the aid of a third party, a consensus was reached. During the selection phase, 25 duplicate articles were removed, leaving 37 for review of titles, abstracts, and keywords. Subsequently, another 24 articles were excluded because they did not relate to financial toxicity, were not conducted during the pandemic, or involved children, adolescents, and caregivers as the sample. This process resulted in a final selection of 13 articles. All selected articles were read in their entirety and included in the analysis.

Following the review, data were extracted, organized, and categorized in the following sequence: authorship, publication year, objectives of the study, and the repercussions of financial toxicity during the COVID-19 pandemic.

To determine the level of evidence of the studies, the classification system from the “Oxford Centre for Evidence-based Medicine: Levels of Evidence” was used^(12)^.

The need for submission to an ethics review board was waived since this was a review study using publicly available literature.

## RESULTS

**Figure 1:**
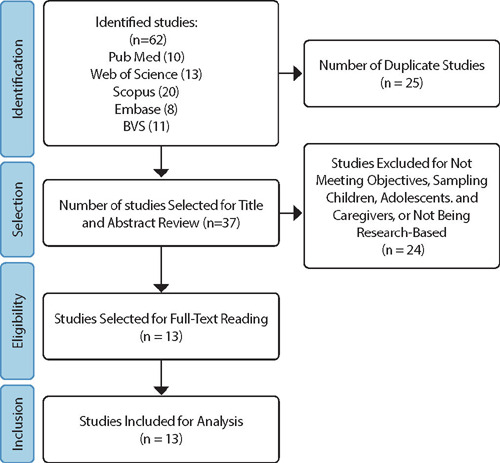
Flowchart of the Selection Process, Curitiba, Paraná, Brazil, 2023

The search yielded 62 studies, with 20 from Scopus, 13 from Web of Science, 11 from the Virtual Health Library (BVS), 10 from PubMed, and 8 from Embase, as depicted in Figure 1

Regarding the publication year and language of the analyzed studies, five were published in 2021, seven in 2022, and one in 2023, all in English. As for the type of approach of the studies, 11 are quantitative and two are qualitative. Concerning the level of evidence, all studies presented a low level - 5. Chart 2 displays the type of study, year of publication, sample, objectives, and the main repercussions of financial toxicity during the COVID-19 pandemic.

**Chart 2:** Synthesis of Studies Included in the Analysis, Curitiba, Paraná, Brazil, 2023

Author(s) / Year of Publication / Country of Study / Healthcare System	Type of Study / Sample	Objective	Repercussion of Financial Toxicity
Staehler et al., 2021^(13)^ USA / Private Healthcare System	- Quantitative Research 539 individuals with renal cell carcinoma	Assess the financial toxicity associated with renal cell carcinoma during the COVID-19 crisis.	23% of patients did not feel in control of their financial situation. Metastatic patients who had not started systemic therapy showed a lower financial toxicity score than those undergoing oral therapy.
Williams et al., 2022^(14)^ USA / Private Healthcare System	- Quantitative Research 1,437 individuals with cancer	Examine the relationship between health insurance coverage and the challenges of covering health-related and non-health-related expenses during the COVID-19 pandemic in adults with cancer.	57% reported challenges in paying for necessities during the pandemic, with 40% having trouble paying for food, 32% for housing, 28% for transportation, and 20% for internet.
Chen et al., 2021^(15)^ USA / Private Healthcare System	- Quantitative Research 100 women with gynecological cancer	Determine the impact of COVID-19 on cancer treatment, anxiety, and financial difficulties among low-income patients with gynecological cancer during the peak of the pandemic in New York.	There was an increase in concern and anxiety about cancer; delays in medical care were reported.
Thom et al., 2021^(16)^ USA / Private Healthcare System	- Quantitative Research 212 young adults with cancer	Describe the financial toxicity experienced during the pandemic; Analyze the relationships between financial toxicity, negative economic events, and cost-coping behaviors; Identify prevalent cost-coping behaviors.	36% stated that their credit card debt increased; 21% reported having no money to pay their rent or mortgage; 19% had no money for medical expenses; 17% for food, and 12% for medication; 19% lost their jobs; 15% reported a reduction in salary.
Zhao et al., 2021^(17)^ USA / Private Healthcare System	- Quantitative Research 2,661 patients with breast cancer	Measure the psychosocial well-being of participants before and during the COVID-19 pandemic, assessing whether they encountered any financial challenges or disruptions in treatment.	One-third of the participants experienced some type of financial challenge during this period. A quarter of the participants had difficulty obtaining treatment.
Wadasadawala et al., 2021^(18)^ India / Mixed Healthcare System	- Quantitative Research 138 patients with non-metastatic breast cancer	Examine the economic hardship faced by breast cancer patients undergoing treatment at the Tata Memorial Center (TMC) in Mumbai, India, during the national lockdown that began in March 2020 following the outbreak of COVID-19.	The average monthly expenditure of cancer patients increased by 32%; the average monthly family income was reduced. More than two-thirds had no income during the lockdown. More than half took out loans; 30% used their savings; 28% received charity; 81% reported a lack of money; 32% reported a shortage of food; 28% reported a shortage of medication.
Hassan et al., 2022^(19)^ USA / Private Healthcare System	- Quantitative Research 627 patients with cancer	Characterize the use of telemedicine among cancer patients facing financial difficulties during the COVID-19 pandemic.	Telemedicine was widely adopted during the pandemic, with most patients preferring video consultations. Telemedicine can reduce existing inequalities, particularly among the vulnerable cancer population.
Peoples et al., 2022^(20)^ USA / Private Healthcare System	- Quantitative Research 1,472 adult patients with cancer	Evaluate the healthcare experiences related to the pandemic, COVID-19 prevention measures, health behaviors, and psychosocial outcomes among rural and urban cancer patients.	Financial stress was common among both rural and urban cancer patients.
Patel et al., 2023^(21)^ USA / Private Healthcare System	- Quantitative Research 11,688 patients with cancer	Estimate the travel, time, and cost savings associated with telehealth for the delivery of oncology care.	Telehealth was associated with time savings and reduced travel costs for patients, which can decrease the financial toxicity of cancer treatment. Expanding oncology telehealth services could be an effective strategy to reduce the financial burden on cancer patients.
Teteh et al., 2022^(22)^ USA / Private Healthcare System	- Qualitative Research 19 individuals with cancer	Explore the impact of COVID-19 on care and quality of life for lung cancer patients.	Isolation and its impact on social support. Psychological suffering. Care impacted and postponed. Financial impact. Minimal impact on oncology care, work situation, income, or housing.
Ludwigson et al., 2022^(23)^ USA / Private Healthcare System	- Quantitative Research 133 patients with breast cancer	Investigate the concerns of breast cancer patients related to COVID-19.	50% of participants reported fear about how the COVID-19 pandemic would affect their care or recovery from cancer; 66% reported anxiety about contracting COVID-19; 22% reported a decrease in income. Interviews provided insights into the advantages and disadvantages of telehealth.
Zomerdijk et al., 2022^(24)^ Australia / Mixed Healthcare System	- Qualitative Research 24 patients with hematologic cancer	Explore the experiences and needs of patients with hematologic cancer during the pandemic.	Fear of contracting COVID-19; behavioral changes to protect health; impact on daily routine and habits; reduction in social support and access to external support services; increased financial difficulties, worsening health.
Kieran et al., 2022^(25)^ Ireland / Private Healthcare System	- Quantitative Research 120 oncology patients	Assess patients’ knowledge of COVID-19 and its impact on their behaviors, concerns, and health experiences.	72% of patients reported health-related concerns, loneliness (51%), and discouragement (42%). Financial toxicity worsened, with an increase in financial concern (78%), a reduction in family income (40%), and increased costs due to the lockdown (62%). Parte superior do formulário Parte inferior do formulário

It was observed that the primary repercussions of financial toxicity included difficulties covering basic expenses such as food^(13,16,18)^, housing^(14,16)^, medication^(16,20)^, transportation^(14)^, and internet^(14)^. There was an increase in anxiety and concerns related to health and financial situations^(15,22-25)^; a reduction or absence of income^(16,18,23,25)^; and various financial challenges such as difficulties, impact, stress, and financial challenges^(13,17,20,22,24)^. Additional issues included difficulty obtaining treatment or accessing healthcare services^(15,17,22-23)^ and reduced social support, impacting routine and lifestyle habits^(22,24)^.

Other noted effects were increased expenses^(18,25)^ and the adoption of telemedicine/telehealth as a less burdensome alternative^(19,21,23)^. Other implications, such as the need for charity^(18)^, indebtedness^(16)^, use of savings^(18)^, job loss^(16)^, loneliness^(17)^, discouragement^(17)^, and worsening health^(16)^, were also identified, albeit to a lesser extent.

## DISCUSSION

This review examined the repercussions of financial toxicity on the lives of adult cancer patients during the COVID-19 pandemic, a period that led to increased expenses, exacerbated the economic crisis, and intensified financial difficulties in this population^(13)^, as well as complicated access to healthcare services^(26)^. A study conducted by a Brazilian foundation^(22)^ revealed that there were 1.7 million fewer hospital admissions over one and a half years of the pandemic compared to the period from January 2018 to June 2019. This reduction was due to the need to reorganize healthcare services to meet the demands of COVID-19, which hindered access to services such as consultations, medications, exams, and scheduled interventions^(27)^, especially among patients with chronic diseases requiring continuous monitoring. Reinforcing this issue, a survey describing the changes in the socioeconomic and health conditions of Brazilians during the COVID-19 pandemic found that 21.7% of the sample sought care, and among these, 13.9% were unable to receive it^(26)^.

Other factors that hindered access to healthcare services included the fear of being infected by the virus and strict adherence to social distancing/isolation measures imposed by authorities. This situation was highlighted by a study^(28)^ conducted by a prominent Brazilian foundation, which found that approximately 75% of Brazilians adhered to social distancing, limiting contact with others and staying home most of the time, thereby reducing the demand for healthcare services for routine/preventive consultations.

The context of social isolation can psychologically affect people^(14)^. Studies estimate that one-third to half of the population may experience psychological and psychiatric consequences if they do not receive adequate care during the pandemic, a situation caused by the loss or reduction of social activities^(29-30)^. The absence of social interaction and dialogue significantly influences people’s routines, especially those with cancer, since relationships share experiences and provide social support. In-person group activities allow dialogue to flow and enable participation in educational and assistance programs that can contribute to self-care and enhance personal self-image.

Thus, cancer patients who strictly adhere to measures imposed by authorities and socially distance themselves from others may experience worsening or the onset of anxiety and depression. Mental health impairment is a recurring outcome in various studies on financial toxicity during the pandemic, and psychological suffering is one of the most noted consequences in the literature^(3)^, as the lack of financial resources to afford treatment often leads to increased anxiety, depression, and a diminished quality of life related to health^(31)^.

In addition to social isolation, mental health can also be impacted by unemployment, a situation highlighted in research conducted by a Brazilian foundation^(28)^ and corroborated by an online study in the USA^(32)^. This study identified financial toxicity among patients with renal cell carcinoma during the COVID-19 pandemic, emphasizing that unemployment exacerbates financial toxicity, primarily by increasing anxiety levels. It also noted that unemployment not only diminishes financial resources and restricts social interactions but also contributes to lowered self-esteem.

In the context of unemployment, a literature review^(33)^ investigating the pandemic’s impacts on financial toxicity among cancer survivors showed that unemployed cancer patients often lose their health insurance. This situation can force patients to choose between undergoing treatment and facing financial ruin for their families, triggering detrimental effects on mental health.

Employment, beyond fulfilling basic human needs, serves as a means of building relationships, exchanging experiences, and achieving personal fulfillment^(10,34)^. A study^(35)^ examining how Brazilian research portrays the “meaning and significance of work” emphasized its vital role in self-actualization and in contributing to the development of human identity. Thus, engaging in work is more than just earning money and sustaining oneself; it represents pride, dignity, facilitates social interaction, and holds a significant place in people’s lives.

Despite changes in work routines due to the COVID-19 pandemic, it is important to note that many jobs are not compatible with remote work and were directly affected, particularly those in the trade, logistics, food, automotive, and aesthetics sectors. Consequently, cancer patients working in these sectors might risk their lives to not lose their source of income or could be forced into temporary unemployment due to fear of virus exposure, intensifying the threat of financial toxicity^(36)^.

The interruption of group activities can also lead to a reduction or termination of emotional relationships, potentially damaging self-esteem and mental health. The presence of a partner can provide security and support, alleviating fear and depressive feelings related to the illness and treatment. Contact with family and friends is crucial in managing the consequences of illness^(31)^ and directly influences the health-related quality of life.

The health-related quality of life of cancer patients during the pandemic can be negatively impacted by the physical, psychological, and social domains that make up the concept of quality of life, all of which are weakened. A study^(37)^ involving women undergoing chemotherapy for breast cancer indicated that psychological disorders, such as anxiety and depression, are linked to decreased quality of life. The research highlighted that work capability is compromised during cancer treatment, with changes in functional capacity that hinder daily activities and social participation, affecting quality of life.

Financial toxicity during the pandemic may have been exacerbated by reductions in patients’ budgets, related to decreased work hours, increased expenses for basic items, and unemployment. An Indian study^(18)^ involving women undergoing breast cancer treatment showed that household income was reduced by a quarter during the COVID-19 pandemic, and patients’ average expenses increased by 32%. It was also observed that more than two-thirds of the patients had no income, and more than half resorted to financial loans.

Regarding the decrease in income, the research with breast cancer patients^(18)^ found that 28% of the sample survived with the help of charity and faced a lack of medication due to a shortage of resources. In this context, telemedicine was highlighted as a quick resource capable of minimizing existing inequalities, particularly among cancer patients in financially vulnerable situations^(13)^.

Another point of concern was highlighted by a study^(16)^ that expressed worry about the allocation of investments to COVID-19 treatment, which could lead, in the medium to long term, to financial consequences in areas such as oncology, affecting, for example, the supply of essential medications. This reality could exacerbate financial toxicity, especially in developing countries like Brazil.

### Study limitations

The limitations of this study can be considered as follows: 1) The analyzed population consisted exclusively of adult cancer patients, thereby excluding studies conducted with children, adolescents, and caregivers of cancer patients; 2) The use of “financial toxicity” as an alternative term in the Health Sciences Descriptors (DeCS) may have reduced the number of studies found on the Virtual Health Library (BVS) portal.

### Contributions to Health Care and Nursing

This review focuses on the dissemination and expansion of understanding regarding this adverse event in oncological treatment. It aims to highlight, through literature, the financial hardships faced by cancer patients during the COVID-19 pandemic. One method to mitigate patient suffering is through dialogue. Healthcare teams must be prepared to discuss treatment costs and provide less burdensome alternatives and/or support. Concurrently, families should seek assistance from these teams and managers and be prepared to discuss personal matters, such as finances. It is crucial that patients, families, healthcare teams, managers, and the industry discuss all difficulties, including financial challenges.

In this context, revealing financial toxicity and its consequences in the life of a cancer patient is essential for health professionals, managers, and patients to grasp the complexity of the issue and collaboratively develop strategies to minimize its effects.

## CONCLUSIONS

Financial toxicity significantly impacted the lives of adult cancer patients during the COVID-19 pandemic in several ways, primarily: difficulties in covering basic expenses, reduction or absence of income, increased expenses, and heightened anxiety. Recognizing financial toxicity as an adverse event that significantly affects people’s lives and can become chronic is necessary. The pandemic context exacerbated these difficulties due to resource reductions. Therefore, healthcare teams and managers need to recognize and understand how these challenges can affect different aspects of life and patient profiles.
