# Supplementary material for: Impact of financial toxicity on adults with cancer during the COVID-19 pandemic: an integrative review
Source: Rev Bras Enferm. 2024 Aug 30;77(Suppl 1):e20240078. doi: 10.1590/0034-7167-2024-0078 (PMC11368384; doi:10.1590/0034-7167-2024-0078)
Supplement: Translation

# Translation: Repercussões da toxicidade financeira em adultos com câncer durante a pandemia da COVID-19: revisão integrativa

**DOI:** 10.1590/0034-7167-2024-0078pt

**Authors:** Luciana de Alcantara Nogueira, Cristiano de Oliveira Ribeiro, Leonel dos Santos Silva, Yasmin Hiorrana dos Santos, Luciana Puchalski Kalinke

**Affiliations:** I Universidade Federal do Paraná. Curitiba, Paraná, Brasil

**Keywords:** Estresse Financeiro, Infecções por Coronavírus, COVID-19, Neoplasias, Revisão

## Abstract

**Objetivos:**

identificar as repercussões da toxicidade financeira na vida de pacientes adultos com câncer durante a pandemia da COVID-19.

**Métodos:**

revisão integrativa realizada nas plataformas de dados PubMed, *Web of Science,* Scopus, Embase e no portal da Biblioteca Virtual em Saúde no mês de março de 2023.

**Resultados:**

de 62 estudos encontrados, 13 foram incluídos para análise. As principais repercussões da toxicidade financeira foram: dificuldades em custear despesas básicas como alimentação, moradia, medicamentos, transporte e internet; aumento da ansiedade e preocupações relacionadas à saúde e à situação financeira; redução ou ausência de renda; dificuldade para obter tratamento ou acesso aos serviços de saúde; aumento das despesas e telemedicina como alternativa menos onerosa.

**Conclusões:**

a pandemia acentuou a toxicidade financeira; portanto, as equipes de saúde precisam reconhecê-la como um evento adverso do tratamento oncológico e compreender que ela pode afetar diferentes âmbitos da vida dos pacientes.

## INTRODUÇÃO

A toxicidade financeira é um evento adverso de doenças onerosas como o câncer, estando atrelada às consequências experimentadas por pacientes que têm dificuldades em custear as despesas provenientes do diagnóstico, tais como exames, medicamentos, transporte e outros custos relacionados. Essa situação expõe os pacientes à sobrecarga psicológica, incluindo preocupações financeiras e questões relacionadas à cobertura do plano de saúde (quando disponível). Esses desafios podem levar à negligência, atrasos ou redução nos cuidados necessários^(1-5)^.

O câncer, dentre as doenças crônicas não transmissíveis, apresenta o maior custo associado ao tratamento. Uma demonstração disso pode ser observada em um estudo realizado nos Estados Unidos da América (EUA)^(6)^, país onde ocorre a maioria das pesquisas relacionadas aos valores das drogas contra o câncer. Esse estudo mostrou que o tratamento com cetuximabe®, utilizado para tratar câncer de pulmão, custa em média US$ 80.000 por mês, o que se traduz em US$ 960.000 para prolongar a vida de um paciente por um ano, situação que pode ocasionar ou agravar a toxicidade financeira.

Outra circunstância que pode ter intensificado a toxicidade financeira foi a pandemia da *Coronavirus Disease* 2019 (COVID-19), visto que ela ocasionou uma crise econômica mundial, com diminuição de renda e adoção de diferentes regras de comportamento que impactaram a vida de toda a população. Neste contexto, o paciente fragilizado pelo diagnóstico e tratamento pode sofrer mais com os efeitos da toxicidade financeira, uma vez que passou a conviver com o medo de se infectar pelo vírus, com a ausência de recursos provocada pelo aumento dos custos com alimentação, medicamentos, privação de contato social, entre outras questões, que podem acentuar quadros de depressão e ansiedade.

Pesquisas sobre a toxicidade financeira em âmbito nacional são incipientes, especialmente no período da pandemia da COVID-19. No entanto, estudos^(7-8)^ publicados em 2020 destacam os impactos da crise econômica associada ao momento pandêmico, com ênfase no desemprego, falência e outros indicadores que podem afetar o Produto Interno Bruto (PIB) de médio e longo prazo e acentuar as dificuldades no custeio do tratamento do câncer. Um editorial brasileiro^(9)^ recentemente publicado enfatizou que foi possível observar que a pandemia criou uma crise de proporções inéditas, em que a população se encontrava em ampla vulnerabilidade social e econômica somada ao colapso do Sistema Único de Saúde (SUS), resultando em sobrecarga financeira aos pacientes para o custeio de despesas do tratamento.

Nos EUA, país pioneiro nas discussões sobre a toxicidade financeira, os encargos financeiros ocorridos durante o período de pandemia impactaram na utilização dos serviços de saúde e dificultaram o acesso aos cuidados em saúde, com piores impactos entre as mulheres^(10)^. Nessa diretriz, o estudo se justifica à medida em que, dada a contemporaneidade da pandemia da COVID-19, pouco se discutiu no Brasil e em outros países sobre as repercussões da toxicidade financeira em pacientes com câncer, tornando a temática inovadora e de relevância para o meio acadêmico.

## OBJETIVOS

Identificar as repercussões da toxicidade financeira na vida de pacientes adultos com câncer durante a pandemia da COVID-19.

## MÉTODOS

Trata-se de uma revisão integrativa desenvolvida em seis passos: 1) identificação do tema e definição da pergunta de pesquisa; 2) estabelecimento da amostra e dos critérios de inclusão; 3) definição das informações a serem extraídas dos estudos; 4) análise dos resultados; 5) interpretação dos resultados; 6) apresentação dos achados^(11)^.

Utilizou-se a combinação mnemônica PCC: P (População) – Pacientes adultos com câncer; C (Conceito) – toxicidade financeira; C (Contexto) – pandemia da COVID-19. Com base nessas definições, a pesquisa foi conduzida com a seguinte questão norteadora: Quais as repercussões da toxicidade financeira em pacientes adultos com câncer durante a pandemia da COVID-19 disponíveis na literatura?

A busca pelos estudos foi realizada em março de 2023 nas bases de dados *National Library of Medicine (PubMed), Web of Science, Scopus, Embase* e no Portal da Biblioteca Virtual em Saúde (BVS). Utilizaram-se descritores contidos na plataforma de Descritores em Ciências da Saúde (DeCS) e no *Medical Subject Headings* (MeSH): “*financial toxicity”, “financial stress”, “neoplasms”* e “COVID-19” e seus termos alternativos “câncer” e “toxicidade financeira”, combinados entre si, utilizando os operadores booleanos *AND* e *OR* (Quadro 1).

**Quadro 1:** Estratégia de busca utilizada, Curitiba, Paraná, Brasil, 2023

Bases consultadas e estratégia de busca utilizada	
*Pubmed*	*“financial toxicity”* OR “*financial stress”* AND “*neoplasms”* AND “*covid-19”*
*Web of Science*	*“financial toxicity”* OR “*financial stress” AND “cancer” AND “covid-19”*
*Scopus*	*“financial toxicity”* OR “*financial stress” AND “neoplasms” AND “covid-19”*
*Embase*	*“financial toxicity”* OR “*financial stress” AND “neoplasms” AND “covid-19”*
BVS	“toxicidade financeira” *AND “*câncer” *AND “covid-19”*

Foram incluídos estudos de acesso aberto que apontassem as repercussões da toxicidade financeira utilizando apenas os descritores mencionados, publicados a partir do início da pandemia (2020) até março de 2023, de cunho quantitativo e qualitativo, nos idiomas português, inglês ou espanhol. Foram excluídos os textos intitulados como comentários, editorial, carta ao editor e pré-print.

A estratégia de busca e os descritores utilizados encontram-se no Quadro 1.

O processo de seleção dos artigos foi realizado por dois pesquisadores independentes para evitar vieses de seleção. Eventuais discordâncias foram discutidas e avaliadas conjuntamente, e com o auxílio de um terceiro, estabeleceu-se um consenso. Durante a seleção, foram excluídos 25 artigos duplicados (idênticos), restando 37 para a leitura dos títulos, resumos e palavras-chave. Após essa leitura, outros 24 foram excluídos por não apresentarem relação com a toxicidade financeira, não terem sido realizados durante a pandemia ou por disporem como amostra crianças, adolescentes e cuidadores, restando, portanto, 13 artigos. Todos foram lidos na íntegra e incluídos na análise.

Após a leitura dos artigos, os dados foram extraídos, organizados e caracterizados na seguinte ordem: autoria, ano de publicação, objetivos do estudo e repercussões da toxicidade financeira durante a pandemia da COVID-19.

Para estabelecer o nível de evidência dos estudos, adotou-se a classificação de Oxford intitulada “*Oxford Centre for Evidence-based Medicine: Levels of Evidence”* ^(12)^.

A submissão ao Comitê de Ética em Pesquisa para análise e aprovação foi dispensada, por se tratar de um estudo de revisão coletado a partir de artigos disponíveis na literatura.

## RESULTADOS

A pesquisa localizou 62 estudos, sendo 20 na Scopus, 13 no *Web of Science,* 11 na BVS, 10 no *PubMed* e oito na *Embase,* conforme Figura 1.

**Figura 1:** Fluxograma do processo de seleção, Curitiba, Paraná, Brasil, 2023

Com relação ao ano de publicação e idioma dos estudos analisados, cinco foram publicados em 2021, sete em 2022 e um em 2023, todos no idioma inglês. Em se tratando do tipo de abordagem dos estudos, 11 são quantitativos e dois qualitativos. Em relação ao nível de evidência, todos os estudos apresentaram baixo nível - 5.

O Quadro 2 expõe o tipo de estudo e ano de publicação, amostra, objetivos e as principais repercussões da toxicidade financeira durante a pandemia da COVID-19.

**Quadro 2:** Síntese dos estudos incluídos na análise, Curitiba, Paraná, Brasil, 2023

Autor (es)/Ano de publicação / país de desenvolvimento / sistema de saúde	Tipo de Estudo/amostra	Objetivo	Repercussão da toxicidade financeira
Staehler et al., 2021^(13)^ EUA Privado	-Pesquisa quantitativa 539 pessoas com carcinoma de células renais	Verificar a toxicidade financeira do carcinoma de células renais durante a crise da COVID-19.	23% dos pacientes não se sentiam no controle de sua situação financeira. Pacientes metastáticos que não iniciaram a terapia sistêmica apresentaram escore de toxicidade financeira menor do que aqueles que usavam terapia por via oral.
Williams et al., 2022^(14)^ EUA Privado	-Pesquisa quantitativa 1.437 pessoas com câncer	Examinar a relação entre a cobertura de seguro de saúde e os desafios de pagamento por necessidades de saúde e não relacionadas à saúde durante a pandemia de COVID-19 em adultos com câncer.	57% relataram desafios no pagamento de necessidades durante a pandemia, com 40% relatando problemas para pagar por comida, 32% por moradia, 28% por transporte e 20% por internet.
Chen et al., 2021^(15)^ EUA Privado	-Pesquisa quantitativa 100 mulheres com câncer ginecológico	Determinar o impacto da COVID-19 no tratamento do câncer, ansiedade e dificuldades financeiras entre pacientes de baixa renda com câncer ginecológico durante o pico da pandemia de Nova York.	Aumento da preocupação e ansiedade com o câncer; Atraso no atendimento médico.
Thom et al., 2021^(16)^ EUA Privado	-Pesquisa quantitativa 212 adultos jovens com câncer	1) Descrever a toxicidade financeira durante a pandemia; 2) determinar as relações entre toxicidade financeira, eventos econômicos negativos e comportamentos de enfrentamento de custos; e 3) identificar comportamentos de enfrentamento de custos	36% afirmaram que a dívida de cartão de crédito aumentou; 21% relataram não ter dinheiro para pagar seu aluguel ou hipoteca; 19% relataram não ter dinheiro para despesas médicas; 17% para alimentação e 12% para medicamentos; 19% perderam o emprego; 15% relataram diminuição do salário.
Zhao et al., 2021^(17)^ EUA Privado	-Pesquisa quantitativa 2661 pacientes com câncer de mama	Medir o bem-estar psicossocial dos participantes antes e durante a pandemia de COVID-19 e examinar se eles experimentaram algum tipo de desafio financeiro ou interrupção do tratamento.	Um terço dos participantes experimentou algum tipo de desafio financeiro durante esse período. Um quarto dos participantes teve dificuldade em obter tratamento.
Wadasadawala et al., 2021^(18)^ Índia Privado e público	-Pesquisa quantitativa 138 pacientes com câncer de mama não metastático	Examinar o sofrimento econômico enfrentado por pacientes com câncer de mama que recebem tratamento no Tata Memorial Center (TMC) Mumbai, Índia, durante o *lockdown* nacional iniciado em março de 2020 após o surto de COVID-19.	O gasto médio mensal de pacientes com câncer aumentou 32%; A renda familiar mensal média foi reduzida; Mais de dois terços não tinham renda durante o *lockdown.* Mais da metade fez empréstimo; 30% usaram suas economias; 28% receberam caridade; 81% relataram falta de dinheiro; 32% relataram escassez de alimentos; 28% relataram escassez de medicamentos.
Hassan et al., 2022^(19)^ EUA Privado	-Pesquisa quantitativa 627 pacientes com câncer	Caracterizar a utilização da telemedicina em pacientes com câncer com dificuldades financeiras durante a pandemia de COVID-19.	A telemedicina foi adotada em alta taxa durante a pandemia, com a maioria dos pacientes preferindo as consultas por vídeo. A telemedicina pode diminuir as desigualdades existentes, particularmente entre a população vulnerável ao câncer.
Peoples et al., 2022^(20)^ EUA Privado	-Pesquisa quantitativa 1.472 pacientes adultos com câncer	Avaliar as experiências de cuidados de saúde relacionados à pandemia, comportamentos e medidas de prevenção da COVID-19, comportamentos de saúde e resultados psicossociais entre pacientes com câncer rural e urbano.	O estresse financeiro foi comum entre os pacientes rurais e urbanos com câncer.
Patel et al., 2023^(21)^ EUA Privado	-Pesquisa quantitativa 11.688 pacientes com câncer	Estimar a viagem, o tempo e a economia de custos dos pacientes associados à telessaúde para a prestação de cuidados oncológicos.	A telessaúde esteve associada à economia de tempo e nos custos de deslocamento dos pacientes, o que pode reduzir a toxicidade financeira do tratamento oncológico. A expansão dos serviços de telessaúde oncológica pode ser uma estratégia eficaz para reduzir a carga financeira entre os pacientes com câncer.
Teteh et al., 2022^(22)^ EUA Privado	-Pesquisa qualitativa 19 pessoas com câncer	Explorar o impacto da COVID-19 na assistência e qualidade de vida de pacientes com câncer de pulmão.	Isolamento e impacto no apoio social. Sofrimento psíquico. Cuidados impactados e postergados. Impacto financeiro. Impacto mínimo nos cuidados oncológicos, situação de trabalho, rendimento ou habitação.
Ludwigson et al., 2022^(23)^ EUA Privado	-Pesquisa quantitativa 133 pacientes com câncer de mama	Explorar as preocupações dos pacientes com câncer de mama relacionadas à COVID-19.	50% dos participantes relataram medo sobre como a pandemia de COVID-19 afetará seus cuidados ou recuperação do câncer; 66% relataram ansiedade sobre contrair COVID-19; 22% relataram diminuição da renda. As entrevistas forneceram informações sobre as vantagens e desvantagens da telessaúde.
Zomerdijk et al., 2022^(24)^ Austrália Privado e público	-Pesquisa qualitativa 24 pacientes com câncer hematológico	Explorar as experiências e necessidades de pacientes com câncer hematológico durante a pandemia.	Medo de contrair COVID-19; Mudanças de comportamento para proteger a saúde; Impacto na rotina diária e nos hábitos; Redução de apoio social e acesso a serviços de apoio externos; Aumento das dificuldades financeiras, piora da saúde.
Kieran et al., 2022^(25)^ Irlanda Privado	-Pesquisa quantitativa 120 pacientes oncológicos	Avaliar o conhecimento do paciente sobre a COVID e seu impacto em seus comportamentos, preocupações e experiência em saúde.	72% dos pacientes relataram preocupação relacionada à saúde, solidão (51%) e desânimo (42%). A toxicidade financeira piorou, com aumento da preocupação financeira (78%), redução da renda familiar (40%) e aumento dos custos devido ao *lockdown* (62%).

Foi possível observar que as principais repercussões da toxicidade financeira foram: dificuldades em custear despesas básicas como alimentação^(13,16,18)^, moradia^(14,16)^, medicamentos^(16,20)^ transporte^(14)^ e internet^(14)^; aumento da ansiedade e preocupações relacionadas à saúde e situação financeira^(15,22-25)^; redução ou ausência de renda^(16,18,23,25)^; repercussões financeiras (dificuldades, impacto, estresse e desafios financeiros)^(13,17,20,22,24)^; dificuldade para obter tratamento ou de acesso aos serviços de saúde ^(15,17,22-23)^; apoio social diminuído com impactos na rotina e nos hábitos de vida^(22,24)^; aumento das despesas^(18,25)^ e telemedicina/ telessaúde como alternativa menos onerosa^(19,21,23)^.

Implicações como necessidade de caridade^(18)^, endividamento^(16)^, uso de economias^(18)^, perda de emprego^(16)^, solidão^(17)^, desânimo^(17)^ e piora da saúde^(16)^ também foram encontrados, no entanto, de forma menos expressiva.

## DISCUSSÃO

Esta revisão verificou as repercussões da toxicidade financeira na vida de pacientes adultos com câncer durante a pandemia de COVID-19, período que resultou no aumento das despesas, no agravamento da crise econômica e na intensificação das dificuldades financeiras nesta população^(13)^, bem como na complexidade para o acesso aos serviços de saúde^(26)^. Um estudo realizado por uma fundação brasileira^(22)^ revelou que, em um ano e meio de pandemia, houve 1,7 milhão de internações a menos em comparação com o período de janeiro de 2018 a junho de 2019. Isso se deveu à necessidade de reorganização dos serviços de saúde para atender às demandas da COVID-19, o que dificultou o acesso a serviços como consultas, medicamentos, exames e intervenções programadas^(27)^, especialmente entre os pacientes com doenças crônicas que necessitam de acompanhamento contínuo. Reafirmando essa questão, uma pesquisa que descreveu as mudanças nas condições socioeconómicas e de saúde dos brasileiros durante a pandemia da COVID-19 encontrou como resultado que 21,7% da amostra buscou por atendimento e, dentre estes, 13,9% não conseguiram^(26)^.

Outras condições que dificultaram o acesso aos serviços de saúde foram o medo de se infectar com o vírus e o cumprimento rigoroso das medidas de distanciamento social/isolamento impostas pelas autoridades. Essa situação foi evidenciada por uma pesquisa^(28)^ realizada por uma importante fundação brasileira, que verificou que aproximadamente 75% dos brasileiros aderiram ao distanciamento social, restringindo o contato com outras pessoas e permanecendo em casa na maior parte do tempo, diminuindo inclusive a procura por serviços de saúde para a realização de consultas de rotina/preventivas.

O contexto de isolamento social pode afetar psicologicamente as pessoas^(14)^. Estudos estimam que de um terço a metade da população pode apresentar consequências psicológicas e psiquiátricas, caso não recebam cuidados adequados durante a pandemia, uma situação causada pela perda ou diminuição das atividades sociais^(29-30)^. A ausência de convívio social, interação e diálogo influencia significativamente a rotina das pessoas, especialmente daquelas com câncer, dado que, por meio das relações, são compartilhadas experiências e oferecido o apoio social. As atividades grupais presenciais permitem que o diálogo flua e possibilitam a participação em programas educativos e assistenciais que podem contribuir com o autocuidado e a valorização da imagem pessoal.

Dessa forma, o paciente com câncer que opta por adotar rigorosamente as medidas impostas pelas autoridades e se distancia socialmente das pessoas pode ter agravamento ou despertar de quadros de ansiedade e depressão. O comprometimento da saúde mental é um resultado recorrente em diversos estudos sobre a toxicidade financeira durante a pandemia, e o sofrimento psicológico é uma das consequências mais apontadas pela literatura^(3)^, visto que a ausência de recursos financeiros para custear o tratamento comumente ocasiona aumento da ansiedade, depressão e diminuição da qualidade de vida relacionada à saúde^(31)^.

Além do isolamento social, a saúde mental pode ser afetada pelo desemprego, situação apontada na pesquisa realizada por uma fundação brasileira^(28)^ e fundamentada por um estudo conduzido *online* ^(32)^ nos EUA, que determinou a toxicidade financeira de pacientes com carcinoma de células renais durante a pandemia de COVID-19. Este estudo reforçou que o desemprego é um agravante da toxicidade financeira, principalmente por elevar os níveis de ansiedade. Eles também destacam que, além de diminuir as condições financeiras e restringir o convívio social, o desemprego contribui para a diminuição da autoestima.

No âmbito do desemprego, a revisão de literatura^(33)^ que investigou os impactos da pandemia na toxicidade financeira entre sobreviventes de câncer mostrou que pacientes com câncer desempregados frequentemente perdem o plano de saúde. Essa situação pode forçar o paciente a escolher entre realizar o tratamento e enfrentar a ruína financeira da família, desencadeando prejuízos à saúde mental.

A atividade laboral, além de possibilitar ao ser humano satisfazer suas necessidades, representa um meio de relacionamento, troca de experiências e realização pessoal^(10,34)^. Nesse sentido, o estudo^(35)^ que analisou como as pesquisas de brasileiros retratam os temas “sentido e significado do trabalho” destacou que ele desempenha um papel primordial na autorrealização e contribui para o desenvolvimento da identidade do ser humano. Assim, exercer uma atividade laboral significa mais do que apenas receber dinheiro e obter sustento, pois expressa orgulho, dignidade, possibilita interação e convívio social e, dessa forma, ocupa um espaço muito importante na vida das pessoas.

Apesar das mudanças na rotina de trabalho adotadas devido à pandemia do novo coronavírus, é importante ressaltar que muitas atividades não são compatíveis com o home office e foram diretamente afetadas, sobretudo aquelas relacionadas ao comércio, ao setor logístico, alimentício, automotivo e estético, entre outros. Assim, pacientes com câncer que atuam nesses setores podem se ver arriscando suas vidas para não perder a fonte de renda ou podem ser forçados ao desemprego temporário pelo medo da exposição ao vírus, intensificando a ameaça da toxicidade financeira^(36)^.

A interrupção das atividades grupais também pode levar à diminuição ou ao término de relacionamentos afetivos, situação que pode trazer danos à autoestima e à saúde mental. A presença de um companheiro(a) pode oferecer segurança e apoio, amenizando o medo e os sentimentos depressivos em relação à doença e ao tratamento. O contato com familiares e amigos é crucial no gerenciamento das consequências do adoecimento^(31)^ e influencia diretamente na qualidade de vida relacionada à saúde.

A qualidade de vida relacionada à saúde do paciente com câncer, durante a pandemia, pode ser impactada negativamente pelos domínios físico, psicológico e social, que compõem o conceito de qualidade de vida e estão fragilizados. Um estudo^(37)^ com mulheres em tratamento quimioterápico para câncer de mama indicou que transtornos psicológicos, como ansiedade e depressão, estão associados à redução da qualidade de vida. A pesquisa ressaltou que a habilidade para o trabalho é comprometida durante o tratamento do câncer, com alterações na capacidade funcional que prejudicam a realização de atividades cotidianas e a participação social, afetando a qualidade de vida.

A toxicidade financeira durante a pandemia pode ter sido acentuada pela redução no orçamento do paciente, relacionada à diminuição da jornada de trabalho, ao aumento das despesas com itens básicos e ao desemprego. Um estudo indiano^(18)^ com mulheres em tratamento de câncer de mama mostrou que a renda domiciliar foi reduzida em um quarto durante a pandemia de COVID-19, e as despesas médias das pacientes aumentaram em 32%. Observou-se também que mais de dois terços das pacientes não tinham renda e que mais da metade recorreu a empréstimos financeiros.

Quanto à diminuição da renda, a pesquisa com pacientes com câncer de mama^(18)^ constatou que 28% da amostra sobrevivia com ajuda de caridade e enfrentava falta de medicamentos por ausência de recursos. Nesse contexto, a telemedicina foi apontada como um recurso rápido e capaz de minimizar as desigualdades existentes, particularmente entre os pacientes com câncer em situação de vulnerabilidade financeira^(13)^.

Outro ponto de atenção foi destacado por um estudo^(16)^ que expressou preocupação com o direcionamento de investimentos ao tratamento da COVID-19, o que poderia acarretar, a médio e longo prazo, consequências financeiras em áreas como a oncologia, afetando, por exemplo, o fornecimento de medicamentos essenciais. Essa realidade pode exacerbar a toxicidade financeira, especialmente em países em desenvolvimento, como o Brasil.

### Limitações do estudo

Podem ser consideradas limitações deste estudo: 1) a população analisada composta exclusivamente por adultos com câncer, excluindo assim os estudos conduzidos com crianças, adolescentes e cuidadores de pessoas com câncer; 2) a utilização da expressão “toxicidade financeira” como um termo alternativo no DeCS, o que pode ter reduzido o número de estudos encontrados no portal da Biblioteca Virtual em Saúde (BVS).

### Contribuições para a área da saúde e enfermagem

As contribuições desta revisão referem-se à divulgação e à ampliação do olhar sobre este evento adverso do tratamento oncológico, visando evidenciar, por meio da literatura, a presença das dificuldades financeiras enfrentadas pelos pacientes com câncer durante a pandemia da COVID-19.

Uma das formas de mitigar o sofrimento do paciente é o diálogo. As equipes precisam estar preparadas para discutir os custos do tratamento e fornecer alternativas menos onerosas e/ou de auxílio. Simultaneamente, as famílias devem buscar ajuda das equipes e gestores e estar aptas para conversar sobre questões pessoais, como as finanças. É imperativo que pacientes, familiares, equipes de saúde, gestores e a indústria falem sobre todas as dificuldades, incluindo as de ordem financeira

Nesse sentido, a divulgação da toxicidade financeira e suas consequências na vida do paciente com câncer é crucial para que profissionais de saúde, gestores e pacientes possam compreender a complexidade da temática e, juntos, encontrar estratégias que minimizem seus efeitos.

## CONCLUSÕES

A toxicidade financeira teve impacto significativo na vida de pacientes adultos com câncer durante a pandemia da COVID-19 de várias maneiras, sendo as principais: dificuldades em custear despesas básicas, redução ou ausência de renda, aumento das despesas e ansiedade, entre outros.

É necessário reconhecer a toxicidade financeira como um evento adverso que impacta significativamente na vida das pessoas e pode se tornar crônico. O contexto da pandemia exacerbou as dificuldades pela diminuição de recursos, desta forma, equipes de saúde e gestores precisam identificar e compreender que ela pode afetar diferentes âmbitos da vida e perfis de pacientes.
